# An image based application in Matlab for automated modelling and morphological analysis of insect wings

**DOI:** 10.1038/s41598-022-17859-9

**Published:** 2022-08-17

**Authors:** Shahab Eshghi, Fatemeh Nabati, Shaghayegh Shafaghi, Vahid Nooraeefar, Abolfazl Darvizeh, Stanislav N. Gorb, Hamed Rajabi

**Affiliations:** 1 Department of Functional Morphology and Biomechanics, Institute of Zoology, Kiel University, Kiel, Germany; 2 Department of Mechanical Engineering, Ahrar Institute of Technology and Higher Education, Rasht, Iran; 3 Faculty of Mechanical Engineering, University of Guilan, Rasht, Iran; 4 Division of Mechanical Engineering and Design, School of Engineering, London South Bank University, London, UK

**Keywords:** 3-D reconstruction, Software

## Abstract

Despite extensive research on the biomechanics of insect wings over the past years, direct mechanical measurements on sensitive wing specimens remain very challenging. This is especially true for examining delicate museum specimens. This has made the finite element method popular in studies of wing biomechanics. Considering the complexities of insect wings, developing a wing model is usually error-prone and time-consuming. Hence, numerical studies in this area have often accompanied oversimplified models. Here we address this challenge by developing a new tool for fast, precise modelling of insect wings. This application, called *WingGram*, uses computer vision to detect the boundaries of wings and wing cells from a 2D image. The app can be used to develop wing models that include complex venations, corrugations and camber. *WingGram* can extract geometric features of the wings, including dimensions of the wing domain and subdomains and the location of vein junctions. Allowing researchers to simply model wings with a variety of forms, shapes and sizes, our application can facilitate studies of insect wing morphology and biomechanics. Being an open-access resource, *WingGram* has a unique application to expand how scientists, educators, and industry professionals analyse insect wings and similar shell structures in other fields, such as aerospace.

## Introduction

Insect wings have various shapes and venation patterns^1,2^. Many studies have aimed to quantify the link between the morphology of the wings and flight performance of insects, including the migratory behaviour^2–4^, aerodynamic force generation^5–9^, territory defence and resource holding^10^, mating behaviour^11^, manoeuvrability and agility^8,12^ and damage tolerance^7,13^. An essential step in this kind of research is to quantify the shape of the wings, their subdomains (known as cells), and the location of the junctions (also known as joints, vein joints or micro joints). However, this is a challenging and time-consuming task, especially for the geometrically complex wings, such as the wings of dragonflies, mayflies, and locusts, which have many wing cells.

In recent years, a few methods have been developed based on deep learning and computer vision to extract the geometric properties of insect wings. *DrawWing*, for example, is an app that extracts the location of junctions of bee wings^14^. A simple developmental model has also been presented recently to extract the venation pattern of insect wings^15,16^. *FijiWings* is another explicitly designed example for studying Drosophila wings^17^. *NET* and *NEFI* are network extraction apps that can extract the location of the junctions^18,19^. All these apps can be used to perform a specific task or for a particular wing type.

Insect wings have also attracted much attention in the field of biomechanics. Considering the technical challenges associated with direct mechanical measurements on insect wings, many studies in this area have used finite element modelling^2,4,20–26^. Finite element software packages, such as SolidWorks, Abaqus and Catia, enable users to develop insect wings models manually. Considering that insect wings are not 2D planar structures but are somewhat wrinkled by intricate patterns of corrugations, which drastically influence their function^27–31^, manual modelling of wings is a time-consuming and error-prone process, even for an expert user. Hence, many previous efforts have resulted in the development of wing models with huge oversimplifications^21,22,24,25,30,32–34^. To the best of our knowledge, the only existing semi-automated tool for finite element modelling insect wings is our previous app, *WingMesh*^35^. However, *WingMesh* represents a significant drawback: the absence of control on the type and size of the produced mesh.

**Figure 1:**
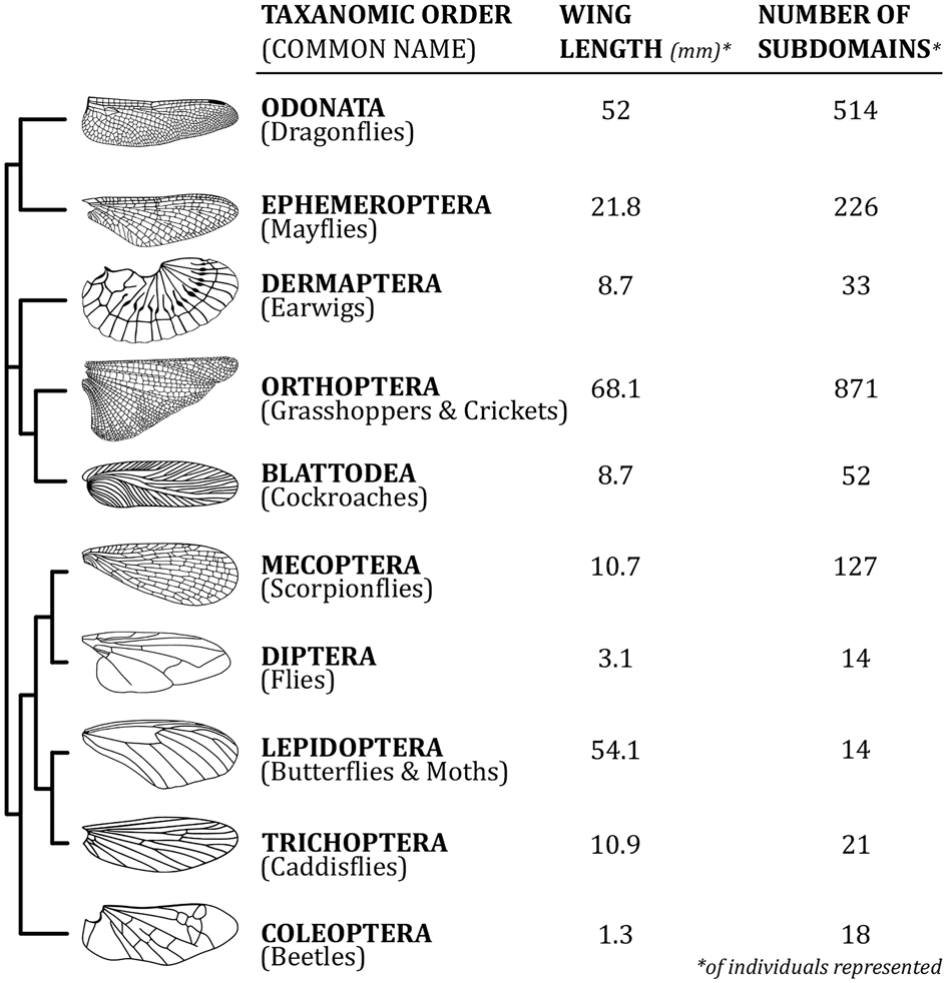
Wings from ten representative insect orders were analysed and modelled using *WingGram* (wing drawings were taken from^16^).

To overcome this methodological gap, here we present a powerful app, *WingGram*, for extracting geometric properties of insect wings and developing precise finite element wing models. We use examples of ten insect wings with different levels of geometric complexity to evaluate the applicability of *WingGram* (Fig. 1).

## Methods and concepts

*WingGram* is an open-access app with a user-friendly graphical user interface (GUI) that we created in App Designer in Matlab 2020b. The fast marching algorithm^36,37^, a well-known method in computer vision, is used to extract the outer edges of an insect wing (here referred to as domain) and those of wing cells (here referred to as sub-domains) using only an input 2D image (“Extracting the boundary of the wing and wing cells”). The Ramer–Douglas–Peucker line simplification algorithm is employed to reduce the saturation of points on the detected boundaries for increasing the runtime (“Ramer–Douglas–Peucker line simplification algorithm”)^38,39^. We improved our previous method, *WingMesh*^35,40^, and included it into *WingGram* to add higher flexibility in modelling 3D corrugations (“Corrugation assignment”). Python scripting in Abaqus is used to generate finite element models (“Generating models using Python scripting in Abaqus”). The fast marching algorithm (“Measurement of geometric properties”) extracts the wing cell's geometric properties. The Zhang-Suen thinning algorithm^41^ is used to skeletonise the lines in an input wing image and detect the location of vein junctions (“Identifying vein junctions”). The finite element simulation from Combes and Daniel^2,4,9^ is used to validate generated model by *WingGram* (“Validation”).

### Extracting the boundary of the wing and wing cells

**Figure 2:**
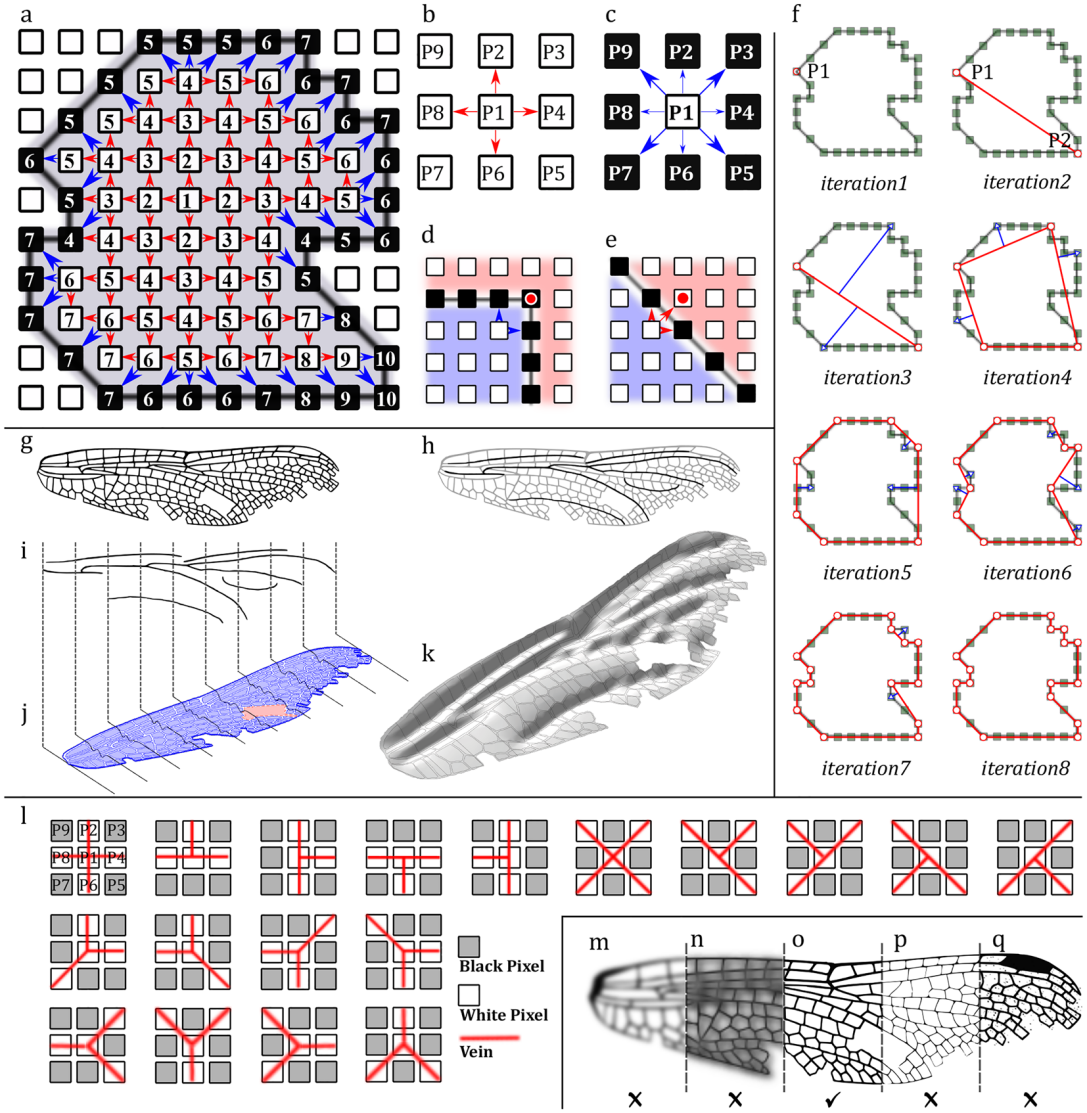
Techniques used in *WingGram*. (**a**) The fast marching algorithm for detecting the boundary of a domain. (**b**) cardinal directions to find new white pixels in the fast marching algorithm. (**c**) All directions to find new black pixels in the fast marching algorithm. (**d**) The fast marching algorithm may miss the point if only the cardinal directions check for finding new black pixels. (**e**) Percolating to neighbour domains may occur if the fast marching algorithm checks all the directions for finding new white pixels. (**f**) Ramer-Douglas-Peucker line simplification algorithm. (**g**–**k**) Assignment of wing corrugations. (**g**) The wing image. (**h**) Highlighted corrugated spots. (**i**) The secondary image (divided into several sections). (**j**) Assignment of sections to the main image. (**k**) The developed corrugated model. (**l**) Defined conditions for the identification of vein junctions. (**m**–**q**) Suitable and non-suitable images. (**m**) fade image. (**n**) Dark image. (**o**) Appropriate image. (**p**) Thin and unclear venations. (**q**) Salt and pepper noise.

The fast marching algorithm is a recursive method in computer vision and detects an arbitrary domain^36,37^. In *WingGram,* we use this algorithm to detect the boundary of insect wings, their wing cells, and any discontinuity within them. Figure 2a illustrates how the fast marching algorithm works. Each square and numbers inside each figure represent a pixel and the iteration phases, respectively. Pixel 1 is an arbitrary white pixel inside the domain. When the first white pixel is selected, the code searches for white pixels around that pixel in the four cardinal directions (i.e. right, left, up and down) (Fig. 2b). When the code finds white pixels around pixel 1, it searches for the neighbour black pixels. However, this time, it checks all eight pixels around pixel 1 (i.e. Fig. 2c, pixels 2 to 9). This strategy distinguishes the neighbour cells, which might not be possible otherwise (Fig. 2d,e). The code stores the coordinates of the found white pixels and uses them as initial pixels for the next iteration. The app also keeps the coordinates of the located black pixels as the domain's boundary. In each iteration, the algorithm changes the colours of the found white and black pixels to light grey and dark grey, respectively, to avoid duplication. A similar procedure continues until there are no undetected white pixels inside the wing.

### Ramer–Douglas–Peucker line simplification algorithm

Using the fast marching algorithm, *WingGram* could detect the pixels located on the boundary of a domain. However, the presence of all these pixels is not required. The higher the number of pixels at the boundary, the higher the computational complexity.

Ramer–Douglas–Peucker line simplification is a recursive method that keeps only the critical points on the boundary and removes the rest^38,39^. This algorithm creates a similar curve, as the original one, with fewer numbers of pixels. In this algorithm, $\varepsilon$ is a criterion of similarity that defines the Hausdorff distance, i.e. the maximum length, between the original curve and the simplified one^42^. A smaller $\varepsilon$ allows more points to remain on the curve. Figure 2f illustrates how this method works on an arbitrary domain. In the first step, this algorithm marks the first and the last points of the curve (the two points overlap in a closed curve). Point P1 in the first iteration is the first found point. Then the algorithm finds the farthest point from the first kept point (Fig. 2f, iteration 2, point P2). If the distance between two found points is more than $\varepsilon$, the algorithm marks and keeps the new point. The algorithm then subdivides the original curve by connecting the two marked points. In each part, the algorithm finds the farthest point from the line between P1 and P2 and marks them as new fixed points if their distance from the line between P1 and P2 is more than $\varepsilon$ (Fig. 2f, iteration 3). The algorithm recursively continues the same procedure for new segments until there are no points in which the distance of the point from the first and the last points of that segment is more than $\varepsilon$ (Fig. 2f, iterations 4 to 8). In the last iteration, only 17 out of 34 initial points remained, without any noticeable change in the curve.

### Corrugation assignment

*WingGram* generates 3D finite element models of an insect wing by modelling wing corrugations. For this purpose, the app needs a secondary image. To make the secondary image, the user can copy the main image, highlight the place of corrugations, and then erase the rest of the image (Fig. 2g–i). The app subdivides the secondary image into several vertical sections (Fig. 2i). In the secondary image, the colour of each pixel represents the height of that pixel (Z coordinate), with white being in the valleys and black in the hills. Like in Eshghi et al.^35,40^, a smoothing method is applied to avoid sudden height changes by introducing a greyish fade margin next to the height maxima. Each section represents a curve(Fig. 2j). When the curves connect, it forms a continuous corrugated plate. Then the boundary of the corrugated plate defines according to that of the main domain in the original input image, which results in a 3D corrugated model (Fig. 2k). See the Supplementary Video S1 to find a visual introduction to preparing the secondary image.

### Generating models using Python scripting in Abaqus

The journal file (i.e., *.jnl file) of Abaqus is a Python script that contains all information regarding the geometry of a generated model. *WingGram* generates 2D-planar and 3D-shell finite element models by adapting the corresponding commands of Abaqus Python scripts. The user can define two sections for all veins and all membranes or multiple sections (““Generate Model and Figures” tab: Generating a *.jnl file”). All required commands to generate any of the two types of models are extracted and embedded in *WingGram*. The app automatically updates the Python scripts to develop a wing model based on the information extracted from an input image. The app uses coordinates extracted from the fast marching algorithm (“Extracting the boundary of the wing and wing cells”) to define veins, membranes, and discontinuities. Python commands regarding the SHELL LOFT tool are used to assign corrugations. For this purpose, at least two loft sections and one loft path are required. The application uses extracted curved sections from the secondary image (“Corrugation assignment”) as loft sections, and a linear path is defined as the loft path. The app uses the boundaries extracted from the image to determine the main domain (wing outer boundary), sub-domain (wing cells) and discontinuities of the final model (“Corrugation assignment”). After importing the model into Abaqus, the user can assign material properties to veins and membranes, set boundary conditions and loading, generate a required mesh and start simulations.

### Measurement of geometric properties

After identifying the wing's geometry, *WingGram* uses the obtained data to quantify wing geometric parameters, including wing cell area, length, and width. The app measures the maximum distance between two points on the boundary of a wing cell as the length. The ratio of the cell area to the cell length is the width of the cell. The cell area is determined using the method described by Bourke for irregular polygons^43^. In this method, *WingGram* considers a closed polygon made up of lines between N vertices $\left({x}_{i} \cdot {y}_{i}\right)$; $i$ is an integer between 0 and $N-1$.

1 $$\frac{1}{2}\sum_{i=0}^{N-1}\left({x}_{i}{y}_{i+1}-{x}_{i+1}{y}_{i}\right),$$

### Identifying vein junctions

To identify the location of the vein junction, i.e. where wing veins intersect, the Zhang-Suen thinning algorithm is used to skeletonise the veins^44^. After the skeletonisation, the width of all lines shrinks to one pixel. The app considers a pixel in the skeletonised image as a vein junction if it meets any conditions illustrated in Fig. 2l. For instance, in the first condition, if P1, P2, P4, P6, and P8 are white and P3, P5, P7, and P9 are black, P1 is a vein junction.

### Validation

To validate the methodology, we developed models of the forewing of the moth *Manduca sexta* and used our model to simulate the mechanical response of the wing to loading. Following the combined experimental and numerical study of Combes and Daniel^4^. we assigned homogeneous Young's moduli of $1.5\times {10}^{8}$ Nm^−2^ and $2.1\times {10}^{12}$ Nm^−2^ to membranes and veins, respectively. The wing was fixed at its base, and a point force of $F = 0.003 N$ was applied to the wing tip. We measured the maximum displacement of the wing and compared that with the experimental and numerical results of the earlier study (see the Supplementary Fig. S3).

## Description of the user interface

**Figure 3:**
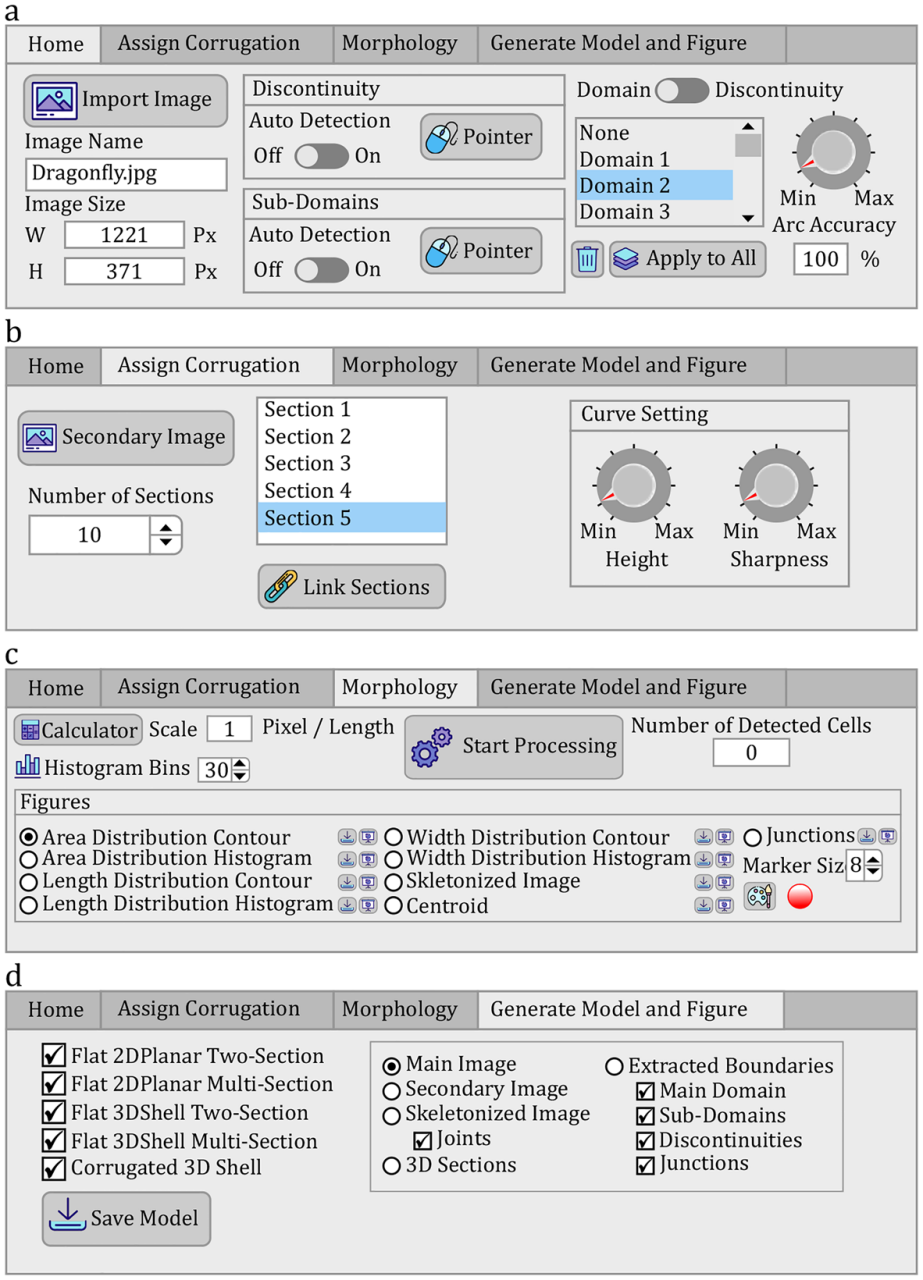
The user interface of *WingGram*. (**a**) This tab is embedded to import the image, specify the discontinuity and subdomains, and change the number of points on the detected boundaries. (**b**) The user can import the secondary image to assign corrugations in this tab. Two knobs are embedded to change the height and the sharpness of the corrugation. (**c**) This tab is embedded to extract the morphological properties of the insect wing. (**d**) Using this tab, the user can save the FE model of the wing. (icons made by Freepik from www.flaticon.com).

The user interface of *WingGram* consists of a tab bar and a display panel. The tab-bar has four tabs, including "Home" (Fig. 3a), "Assign Corrugation" (Fig. 3b), "Morphology" (Fig. 3c), and "Generate Model and Figure" (Fig. 3d). Below the tab bar, a display panel is embedded to show the ongoing processes (see Supplementary File S1 for the installable executive file of *WingGram*). The user instruction and visual description of *WingGram* are available in the Supplementary Video S1.

### "Home" tab: importing an image and detecting discontinuities and sub-domains

The "Home" tab is embedded to import an image of the wing (Fig. 3a). Here we imported an image of a dragonfly wing containing discontinuities. *WingGram* supports non-vectorized *.tif, *.jpg, and *.png image formats. A very high resolution or a binary image is not required. There is no limitation on the size and resolution of the imported image. The position, orientation, and angle of the wing in the image do not influence the applicability of the app. Figure 2m–q shows the criteria for a suitable input image. Faded and dark images or those with salt-and-pepper noises, as shown in Fig. 2m,n,q, influence the performance of the fast marching algorithm in identifying the subdomains. *WingGram* works particularly well if the venation patterns are clear. The application is not ideal for analysing and modelling wings with dark spots or strong pigments. Figure 2p shows thin lines in the input image, which can disturb the performance of the fast marching algorithm for differentiating neighbour subdomains. Figure 2o shows an example of a suitable image. After importing the image, the image's name and size appear in specific fields below the "Import Image" push button (Fig. 3a). By importing the image, the code automatically detects the outer boundary of the wing. In the "Discontinuity" panel, the user can mark the place of discontinuities by pushing the "Pointer" button. Activating the "Auto Detection" switch allows automatic detection of sub-domains and discontinuities. Mark a domain as the discontinuity turns its colour into dark grey. The same procedure is required to detect sub-domains using the "Sub-Domains" panel. Mark a domain as the sub-domain turns its colour into light grey. The next step is to reduce the density of points on the boundary of domains and subdomains. The user can see a list of domains in the "Home" Panel. A blue line appears around it in the display panel by clicking on a domain. Turning the knob-pitch on the right side of the list changes the $\varepsilon$ regarding the Ramer–Douglas–Peucker line simplification method to increase or decrease the density of points (“Ramer–Douglas–Peucker line simplification algorithm”). Red points on the boundary of selected domains show the remaining points after reducing the density. Reducing the number of points on the border reduces the runtime. In this panel, "Arc Accuracy" shows the similarity between the curve of decreasing points and the original curve extracted from the burning algorithm.

Also, there are two buttons under the list. The one with a bin icon is for deleting a domain, and the other one lets the user apply the same node density for all other domains. The same procedure is available for discontinuities. The user can switch between the list of domains and discontinuities at the top of the list.

### "Assign Corrugation" tab: importing the secondary image to assign corrugations

Figure 3b shows the "Assign corrugation" tab bar. The user imports the secondary image using the "Secondary Image" button (“Corrugation assignment”). A spinner is embedded to change the number of sections. We embedded two knobs in our app to change the maximum height (i.e. peak) and sharpness of corrugations. To apply changes to single sections, the user can click on a section, turn the "Link Sections" off, and then use the knobs to adjust each maximum height and sharpness parameter.

### "Morphology" tab: extracting the location of vein junctions and other geometric parameters

Figure 3c shows the "Morphology" tab for extracting the geometric properties of insect wings. To use this panel, the user has to set the scale (i.e., length per pixel) and the number of the histogram bins and then push the "Start Processing" button to start extracting geometric properties from the image of the wing. As described in “Measurement of geometric properties”, the result appears in several figures after the processing ends. The user can switch between the radio buttons to observe each result. Switching between the radio buttons allows visualisation of the contours/histograms of the wing cells' area, length, and width. Other radio buttons show the distributions of the vein junctions, the wing centroid's location, and the skeletonised image. On the right side of each radio button, there are two buttons. One of them saves the data of the corresponding radio button. It keeps the corresponding matrix for contours, the data of histograms, the coordinate of junctions, and the coordinate of the centroid. The second button opens the corresponding figure in a separate window.

### "Generate model and figures" tab: generating a *.jnl file

We embedded four options for developing a planar model without corrugations. The last option works if corrugations are assigned.

- Two-/multi-section, three-dimensional shell: These models are suitable for applying out-of-plane loadings, such as uniform pressure, impact, out-of-plane point force, and displacement^45^.
- Two-/multi-section, two dimensional planar: These models are suitable for applying in-plane loadings, such as shear, tensile, or compressive loading^45^.

Figure 3d shows a group of available figures. Switching between radio buttons shows figures regarding the selected option. On the "Skeletonised Image" button, the user can hide the presence of junctions. On the "Extracted Boundaries" button, the user can mark the presence of the "Main Domain," "Sub-Domains," "Discontinuities," and "Junctions" by turning off corresponding checkboxes.

## *WingGram* in application

**Figure 4:**
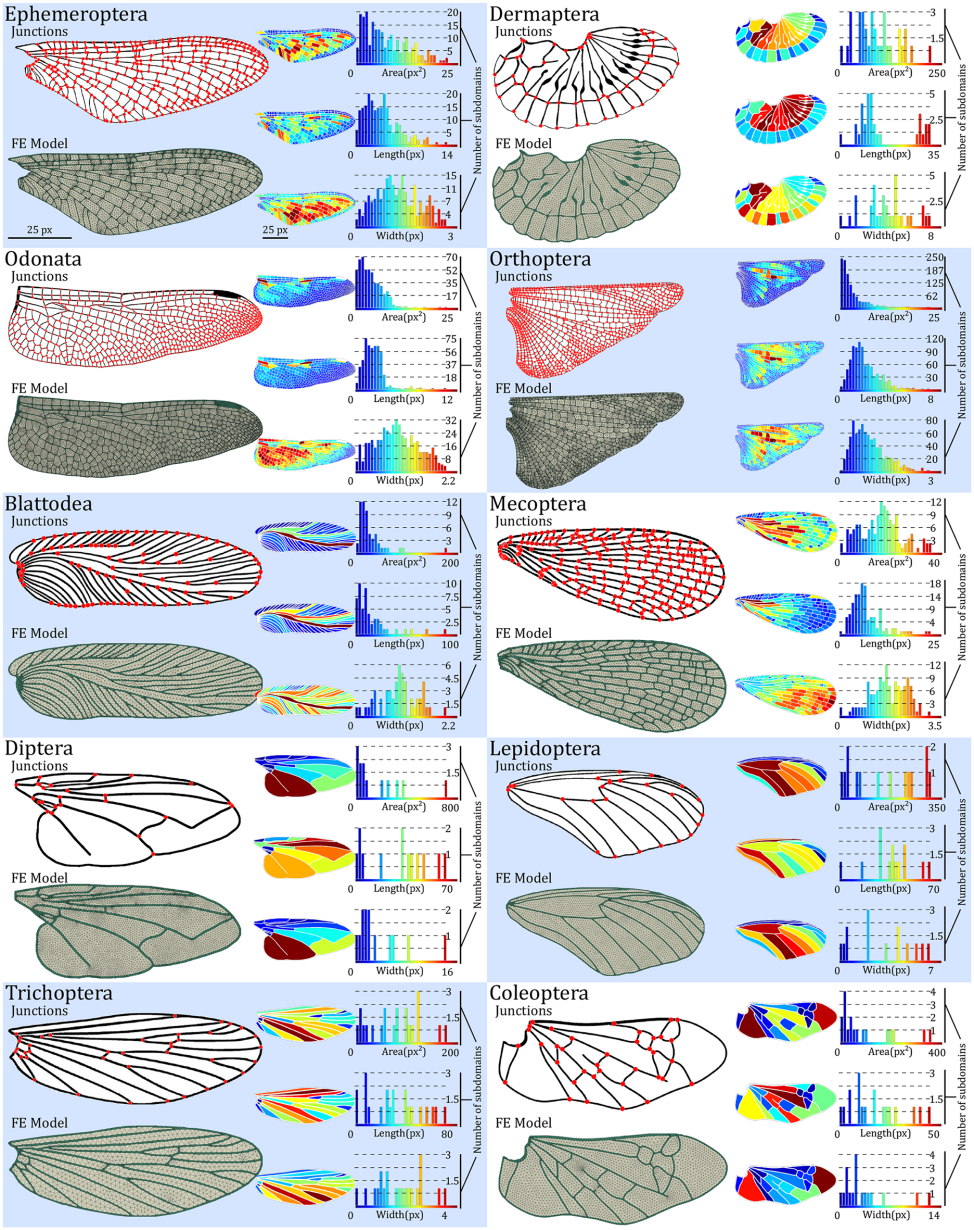
The performance of *WingGram* for extracting the geometric characteristics of ten representative wings from different insect orders. For each wing the FE model, location of vein junctions, area, length, and width distribution contour and histogram are shown. The length of all wings is considered with the same size as 100 pixels. For each wing, three histograms illustrate the distribution of the area (px^2^), length (px), and width (px). Histograms are accompanied by their corresponding contours on the left side. The colour bar of each contour is under its corresponding histogram. The place of junctions are extracted for each wing, and the FE model generated by WingGram is meshed in Abaqus. Insect orders are Ephemeroptera, Dermaptera, Odonata, Orthoptera, Blattodea, Mecoptera, Diptera, Lepidoptera, Trichoptera, and Coleoptera.

Ten representative wing images from different insect orders that had noticeably different shapes and venation patterns were selected to show the performance of *WingGram*, (Fig. 1). Wings were scaled to have the same length as 100 pixels. Figure 4 illustrates the outcome of the *WingGram* for each wing, including the wing's FE model, the vein junctions' location, the distributions and histograms of the cell area, cell length, and cell width (see Supplementary Model S1–S10 for FE models).

**Figure 5:**
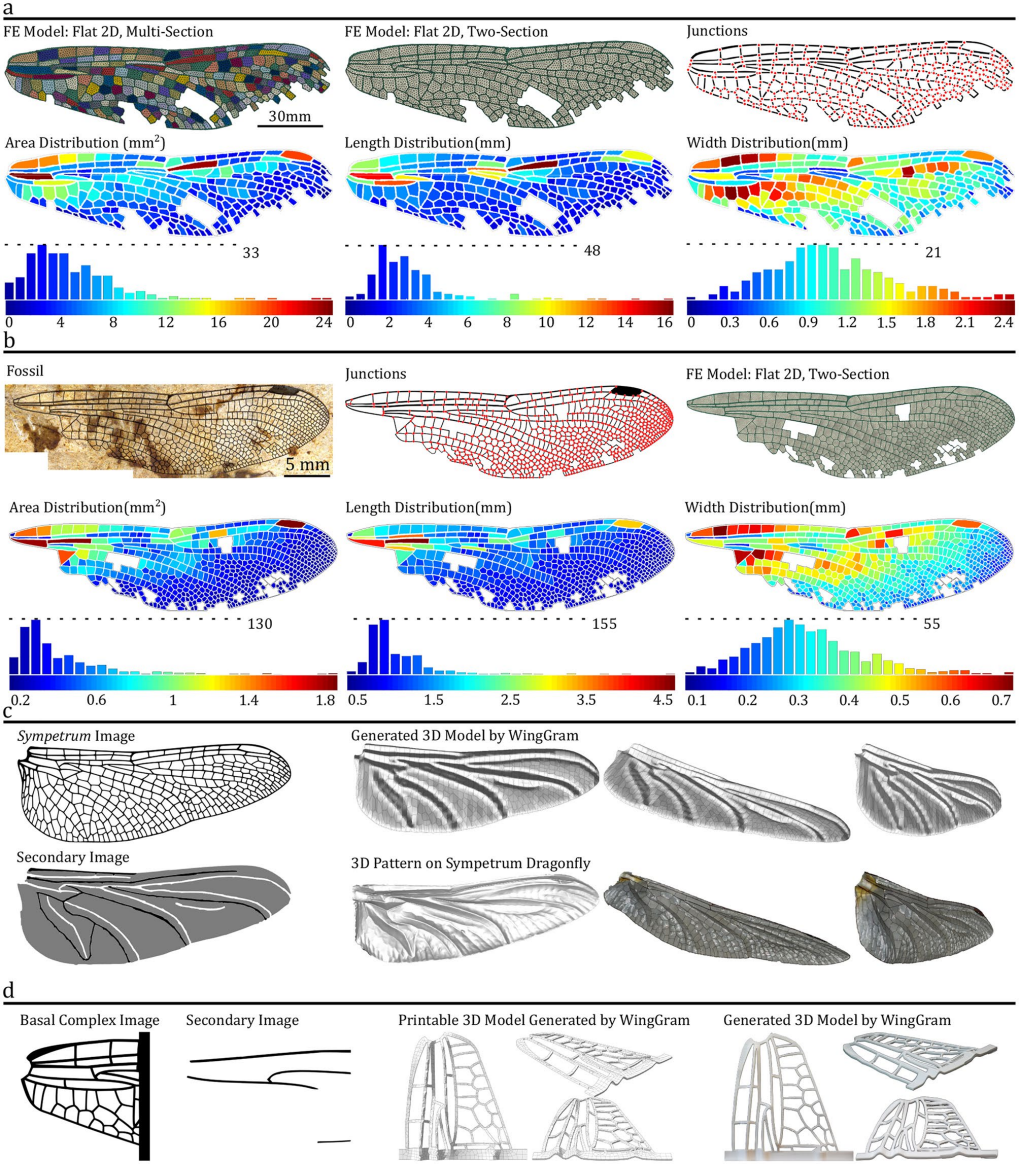
The application of *WingGram* in modelling damaged wings, fossil wings and corrugated wing corrugations, and its use in 3D printing. (**a**) Damaged wing of dragonfly^47^: main image; secondary image to assign corrugation; Flat 2D, two-section FE model; Flat 2D multi-section FE model; corrugated 3D model; location of vein junctions; area, length, and width distribution contour and histogram. (**b**) The fossil wing: photograph of *Auroradraco eos*^46^; location of vein junctions; Flat 2D, two-section FE model; area, length, and width distribution contour and histogram. (**c**) Comparison of the corrugations of a dragonfly wing (*Sympetrum vulgatum*) obtained by a 3D measurement microscope Keyence VR 3100 and *WingGram*: the image of a *Sympetrum* dragonfly; secondary image for assigning corrugations; the 3D model generated by *WingGram*; 3D scan by Keyence VR 3100. (**d**) The 3D printable model generated by *WingGram*: dragonfly basal complex image; secondary image to assign corrugation; the virtual model of the basal complex; the virtual corrugated model developed by WingGram; 3D printed model of the basal complex.

We also anticipate *WingGram* to apply to studies of damaged wings and fossils. Figure 5a,b show a damaged dragonfly wing and the fossil of *Auroradraco eos*^46^. Here we used *WingGram* to extract the location of the vein junctions and the area, length and width of the wing cells for both wings. Also, Using *WingGram*, we developed a geometric multi-section model of the damaged wing and a two-section model of the damaged wing and the fossil (find FE models in the Supplementary Models S11–S13). The models are imported into the Abaqus CAE and are meshed by triangular shell elements. In the multi-section model, we assigned different material properties to the cells. The 3D model of the damaged wing using the secondary image is generated (find the FE model in the Supplementary Model S14).

Assigning corrugations to a wing model in *WingGram* is convenient but might not be very accurate as the user sets them. In Fig. 5c, we used a scanning 3D measurement microscope Keyence VR 3100, to show the 3D pattern on a *Sympetrum* dragonfly hindwing. This image shows the main and secondary image of the wing and the 3D model generated by *WingGram* (find the FE model in the Supplementary Model S15). Also, two other secondary images are used for this model to show how the user can add curvature to the whole model, which is available in Supplementary Fig. S2.

*WingGram* is a modelling app, and virtual models generated by it could be 3D printed to construct physical models. Figure 5d shows the main and secondary image of the basal complex of the dragonfly forewing, the virtual model, and the 3D printed model. Due to the limited print area, we isolated the wing model's basal part and removed the thin membranes (See the Supplementary Model S16 for the G-Code). The isolated part of the wing was fabricated using a Prusa i3 MK3S (Prusa Research s.r.o., Prague, Czech Republic) with white coloured polylactic acid (PLA) filament (Prusa Research s.r.o., Prague, Czech Republic).

*WingGram* is also applicable to many 2D natural and artificial structures, such as leaves, spider nets, geographic maps and/or industrial plates and shells (see the Supplementary Fig. S1).

### Validation result

Figure S3 shows the result of the FE simulations. The maximum bending displacement of our model is 5.47 mm in the z-direction, which is about 4% different from that obtained by Combes and Daniel^4^. The generated model in Abaqus, with its assigned boundary conditions, material properties, and loading, is available in Supplementary Model S17.

## Discussion: limitations of *WingGram* and its advantages over other apps

*WingGram* equips the user with a combination of tools that can also be found in various other apps. We have also included additional unique options to *WingGram*, such as semi-automated finite element modelling. Here, we compare *WingGram* with some of the existing apps.

### WingMesh

*WingMesh* is a Matlab-based app developed by the authors with a user-friendly interface for finite element modelling of insect wings^35^. *WingMesh* can generate both 2D-shell and 3D-corrugated shell models of an insect wing. It can include several sections with different material properties and thickness values for the cells in a wing model. Several features make *WingGram* more powerful than *WingMesh*. The meshing process in *WingMesh* cannot be adjusted and often requires a high runtime. Modifying a model developed by *WingMesh* is not convenient and requires significant effort. *WingMesh* uses *Distmesh2D*, a well-known meshing tool in Matlab^48^, and computer vision to mesh a wing model. The user does not control the type and size of the mesh. The model is not manipulatable in Abaqus because it is made up of orphan meshes. Whilst *WingGram* uses Python scripting in Abaqus to generate JNL models. After importing the model in Abaqus, the user can use all meshing tools to create an ideal mesh in the model and manipulate the geometry easily. The modelling process in *WingMesh* is drastically slower than *WingGram* because the user must wait for Matlab to generate a mesh in the geometry that might not be so precise. *WingMesh* has difficulty in modelling complex wings like dragonfly wings. Because WingGram is more powerful than *WingMesh* and does not abandon any types of insect wings, it has several additional tools which were mentioned earlier in the text. See the Supplementary Fig. S4 for a comparison between modeling a similar wing with *WingGram*, and *WingMesh*. The modeling process from *WingMesh* took about 6 h while the same wing needed only 15 min to be generated by *WingGram*.

### DrawWing

*DrawWing* extracts the location of the vein junctions in an insect wing^14^. This app needs a high-resolution image (more than 2400 dpi × 2400 dpi). As mentioned by its developers, *DrawWing* only works with honeybee wings (*Apis mellifera*). In contrast, *WingGram* does not have any limitations regarding image resolution and is applicable for almost all types of insect wings.

### *NEFI* & *NET*

These apps extract network data from an image using image processing and computer vision. They are applicable for leaf venation, spider webs, crack paths, insect wing venations, and similar structures. They extract the location of junctions and the connection between them, which is applicable for detecting the location of vein junctions of an insect wing^18,19^. This is the only common function between the *NEFI*, *NET,* and *WingGram*. Our application works as accurate as NEFI and NET.

### FijiWings

*FijiWings* uses the advantages of the ImageJ Fiji to measure some geometric features of the Drosophila wing, like the area of the wing and trichome density. This app needs a high-resolution image to work appropriately^17^. In contrast to *FijiWings*, *WingGram* extracts the area, length, and width of subdomains, even if there are damaged input wings, and is applicable for almost all insect wings.

*WingGram* contains some unique features applicable to field studies on insect wings regarding the wing's mechanical behaviour or morphological properties. To the best of our knowledge, *WingGram* is the only existing tool that can be used to develop a precise geometric model (not only mesh) using only an image of insect wings. *WingGram* can facilitate future studies on the insect wings and their components, such as ambient-, cross-, and longitudinal veins, corrugations, and membranes. For example, the location of junctions can be used to test fluctuating asymmetry between left and right wings, which is important for ecological studies to monitor environmental pollution^49,50^. Also, the wing shape and vein junctions' location can be used to identify insect species^14,51,52^. Extracting the area, length and width of membranes is another feature of *WingGram* that can enable us to study the relationship between wing form and design.

Although compared with many existing modelling methods, *WingGram* offers better efficiency and lesser computational time; it represents some limitations that can still be improved. *WingGram* models wings with homogenous thickness, while the thickness of different parts of the wing can vary from one place to another. *WingGram* does not model the cross-section of veins as they are in reality. The authors are currently working on developing a new version of WingGram, which can overcome these limitations and add more features to it.

## Supplementary Information

Supplementary Figure S1.

Supplementary Figure S2.

Supplementary Figure S3.

Supplementary Figure S4.

Supplementary Information 1.

Supplementary Information 2.

Supplementary Information 3.

Supplementary Information 4.

Supplementary Information 5.

Supplementary Information 6.

Supplementary Information 7.

Supplementary Information 8.

Supplementary Information 9.

Supplementary Information 10.

Supplementary Information 11.

Supplementary Information 12.

Supplementary Information 13.

Supplementary Information 14.

Supplementary Information 15.

Supplementary Information 16.

Supplementary Information 17.

Supplementary Information 18.

Supplementary Video S1.

## Data Availability

All 3D models, figures, and data shown in this study are available in the figshare repository, https://doi.org/10.6084/m9.figshare.13350629.

## Code availability

*WingGram* is made in Matlab Appdesigner. The Matlab file is available at https://doi.org/10.5281/zenodo.6591786.
