# Supplementary figures and images for: An image based application in Matlab for automated modelling and morphological analysis of insect wings
Source: Sci Rep. 2022 Aug 17;12:13917. doi: 10.1038/s41598-022-17859-9 (PMC9386019; doi:10.1038/s41598-022-17859-9)

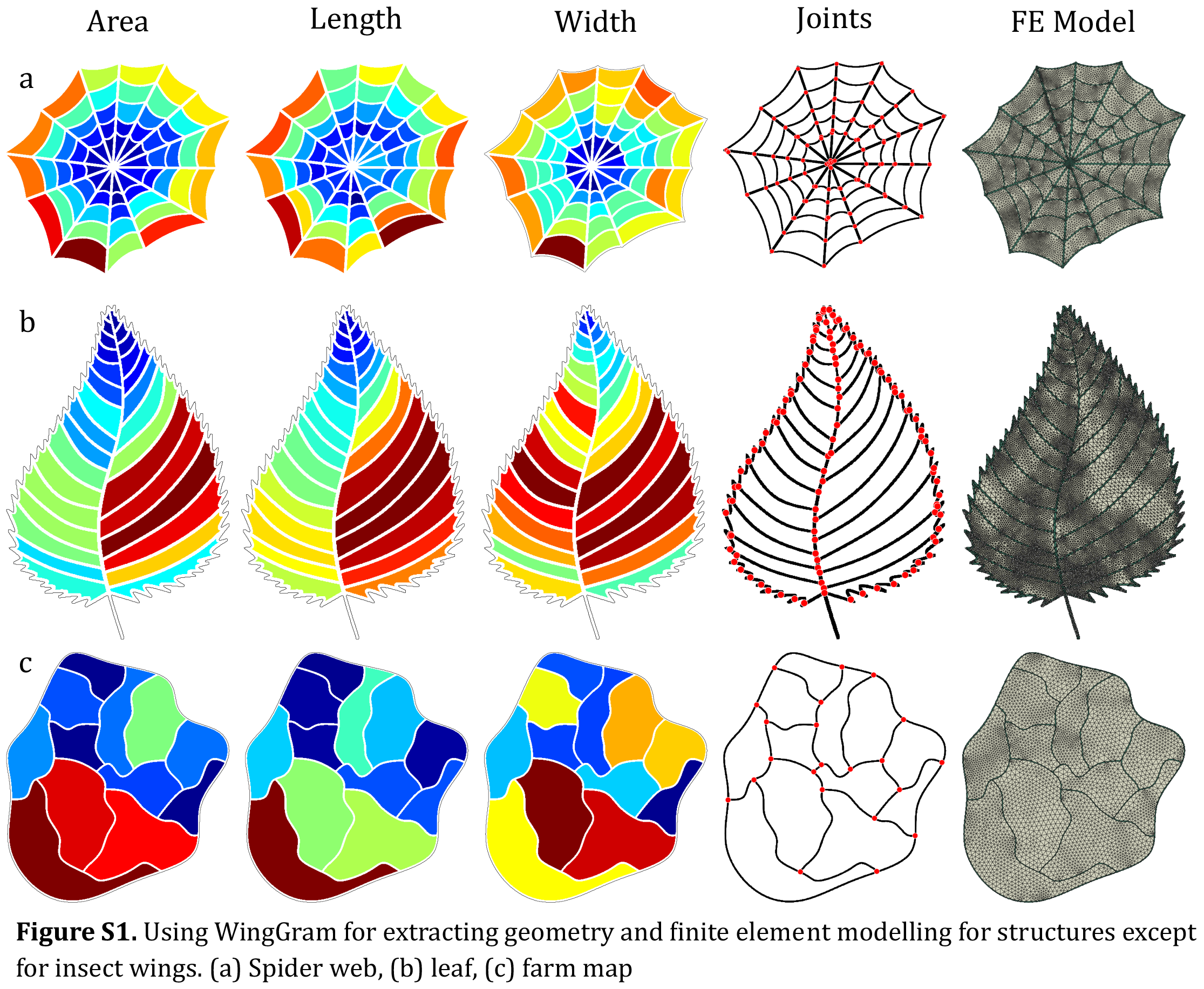

Supplement: Supplementary file 1 — Supplementary Figure S1. [file 41598_2022_17859_MOESM1_ESM.tif]

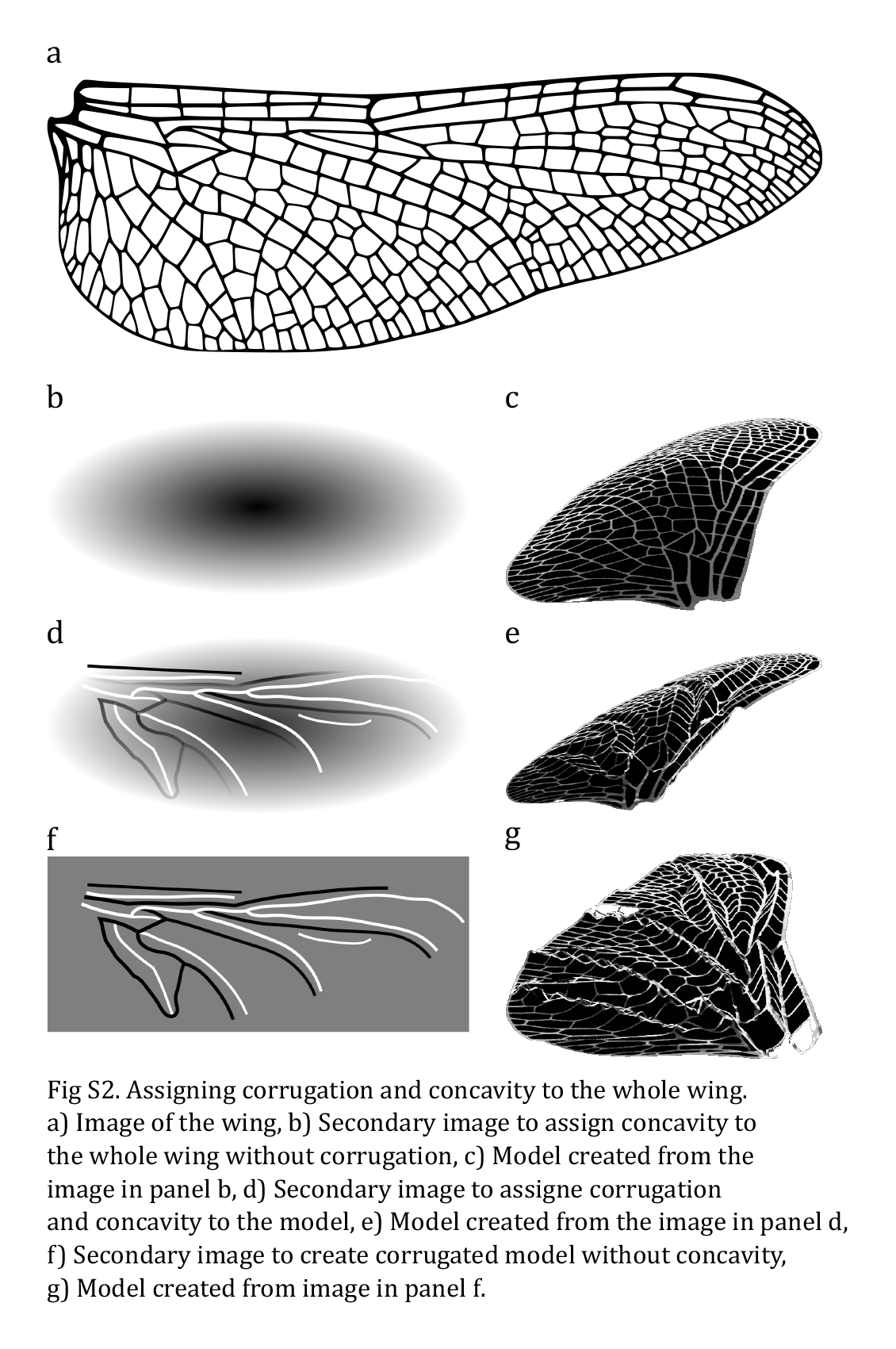

Supplement: Supplementary file 2 — Supplementary Figure S2. [file 41598_2022_17859_MOESM2_ESM.tif]

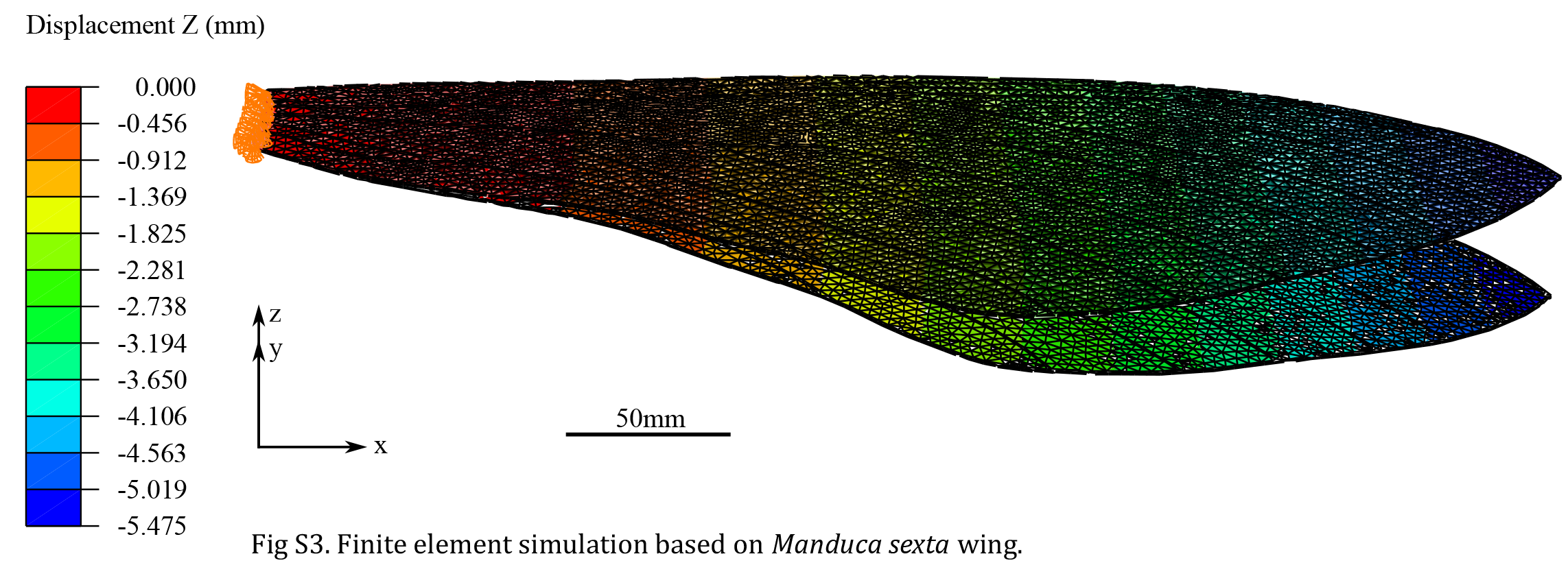

Supplement: Supplementary file 3 — Supplementary Figure S3. [file 41598_2022_17859_MOESM3_ESM.tif]

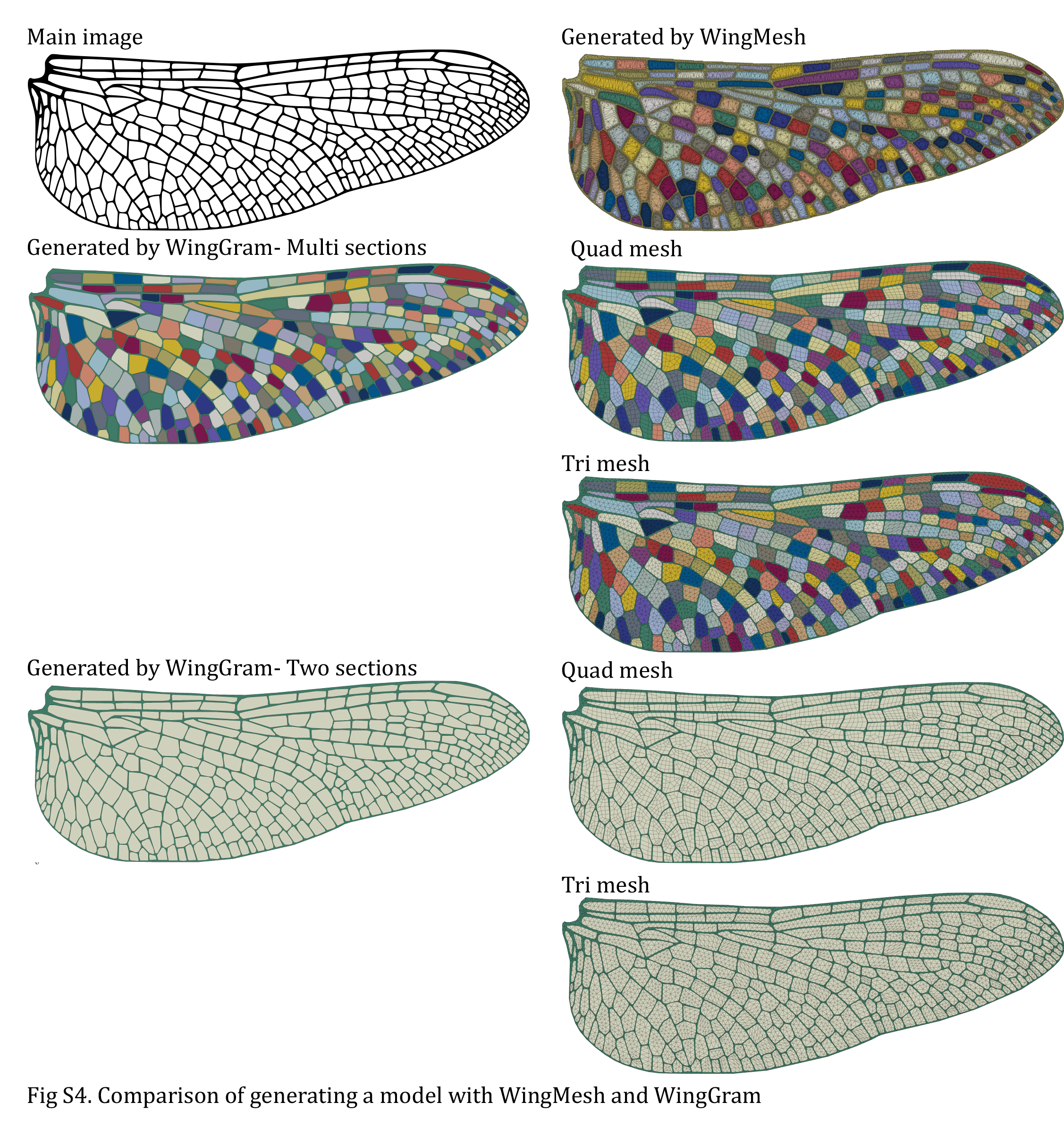

Supplement: Supplementary file 4 — Supplementary Figure S4. [file 41598_2022_17859_MOESM4_ESM.tif]
